# Shortcomings of food-based dietary guidelines worldwide: a focus on sustainability and food processing issues

**DOI:** 10.3389/fnut.2026.1901940

**Published:** 2026-09-18

**Authors:** Anthony Fardet, Marlene Ohlau

**Affiliations:** 1 INRA, UMR 1019, UNH, CRNH Auvergne, F-63000 Clermont-Ferrand & Clermont Auvergne Université, Université d'Auvergne, Unité de Nutrition Humaine, Clermont-Ferrand, France; 2 TMG—Töpfer, Müller, Gaßner GmbH, EUREF-Campus, Berlin, Germany

**Keywords:** Food-based dietary guidelines, shortcomings, sustainability, food processing, holistic approach, recommendation hierarchy

## Abstract

Since 1992, food-based dietary guidelines (FBDGs) have been developed worldwide to help consumers and society on a larger scale make informed food choices for their health. However, the global prevalence of chronic diseases continues to increase, raising questions about the effectiveness of FBDGs in addressing the broader determinants of health, and in promoting sustainable food systems. Therefore, this policy and practice review intends to identify and summarize the main shortcomings of the current FBDGs. The more detailed focus on sustainability and food processing issues showed that these two dimensions are still lacking or insufficiently considered in current FBDGs, hindering the formulation of robust recommendations for protecting global health. Based on this analysis, we propose several paradigms shifts to increase the relevance and coherence of future FBDGs: moving from food pyramids to technological pyramids; shifting from the currently excessive quantitative and food group-based recommendations toward more qualitative and holistic guidance; emphasizing the role of the food matrix rather than isolated nutrients; and integrating sustainability and regenerative agricultural practices more explicitly. The overarching challenge lies in designing FBDGs that adopt a systems-oriented, holistic approach—one that reflects the interconnectedness of human, environmental, and societal wellbeing, and that organizes and hierarchizes nutritional information from broad dietary patterns down to specific nutrients.

## 1 Introduction

The first food-based dietary guidelines (FBDGs) were published in the USA in the 1990s and were notably based on the very first food pyramid (1992) (1). In 1998, a joint FAO/WHO consultation published a report for the preparation and use of FBDG, but it remained closely linked to a nutrient-centered scientific approach (2). Thereafter, several other countries developed FBDGs, *e.g*., the French PNNS (Programme National Nutrition Santé) in 2001. The primary purpose of FBDGs was to shape national nutritional policy for populations of different ages. Initially, these guidelines provided general recommendations designed to ensure nutrient adequacy and prevent chronic diseases. Over time, these guidelines evolved to include more specific advice, such as daily and/or weekly recommended portions for major food groups (3). However, the integration of sustainability (4) and degree of food processing (5) considerations has remained very limited.

Concerning human health, in France, during the 2001–2021 period of the four first PNNS, the prevalence of hypertension, obesity, type 2 diabetes and hepatic steatosis more than doubled (6). Similar observations were made in other developed countries (7). Therefore, despite their regular evolution, one can hypothesize that FBDGs worldwide do not seem to have significantly contributed to slowing the global epidemic of chronic diseases. Therefore, one of the main questions is whether this failure lies with consumers, who may not follow the guidelines, their implementation or with the chosen scientific and academic approach for developing them. In this policy and practice review, we mainly adopt the latter perspective, focusing on the structural and conceptual limitations within the FBDGs themselves.

For example, some well-known recommendations of FBDGs have indirectly played into industry interests more than those of consumers because they provided incomplete and too reductionist messages, *e.g*., “*Eat 5 fruits and vegetables a day”, “Eat, move, it's healthy”*, and “*Avoid eating too sweety, salty and fatty foods*”. A common shortcoming of these slogans is that they do not consider the degree of food processing (8). If they considered food processing, the slogans might have been “*Eat 5 raw or minimally processed fruits and vegetables a day” “Eat raw/minimally processed foods, move, it is healthy*”, etc. Indeed, large beverage companies currently use this last slogan to their advantage because they emphasize that if an individual performs a sufficient amount of physical activity, regardless of the quality of calories consumed, they will not develop chronic diseases and are absolved of responsibility for their unhealthy products (9). They sponsor numerous global sporting events, especially those viewed by the youngest members of the population, to achieve this goal (10).

Here, we have suggested the need for a paradigm change in food and nutrition sciences to elaborate more efficient, qualitative and simpler FBDGs. The main objectives are to identify the main reported shortcomings of FBDGs, and then to focus on two main scientific and emerging issues that have been underestimated in FBDGs, *i.e*., the sustainability issue and degree of food processing, and hypothesized that these issues are potentially the most impactful implementations to perform in the near future for both human and planetary health.

## 2 Methodological framework

This work was carried out as a classic literature review to identify and discuss FBDG shortcomings, focusing on those most important for improving global health. It is part of the European PLAN'EAT project, specifically the sub-section entitled' *Framework for the development of dietary guidelines'* ('*Food Systems Transformation toward Healthy and Sustainable Eating behavior'*, work programme HORIZON-CL6-2021-FARM2FORK-01-15). Following a first consultation with project partners to identify the main FBDG shortcomings, INRAE was primarily responsible for considering the integration of food processing into FBDGs and, secondarily, the sustainability issue.

The literature search to extract publications and international reports was conducted as for a narrative review, using keywords in relation to the topic of this paper (*i.e*., [“guideline^*^ or recommendation^*^] and [food^*^ or diet^*^]) *via* the Web of Science databasis (Clarivate), because it aggregates data from several sources, particularly in the fields of agricultural and food sciences and human health. Concerning reports from international institutions, the search was completed by using Google Scholar.

The initial literature search was conducted in November, 2024, and resulted in an initial European PLAN'EAT internal document written by INRAE and entitled: “*FBDG: shortcomings, sustainability and food processing*”. Only English documents were considered and all countries were taken into account. No other exclusion criteria were applied.

## 3 Assessment of FBDG shortcomings, options and implications

Despite rather shared nutritional goals, FBDGs tend to vary from one country to another, reflecting a different analysis of the scientific literature, different levels of details, and therefore different scientific positions, as notably reported for European countries (11).

Beyond just the European zone, commonly reported FBDG shortcomings have been grouped into six generic topics (Figure 1), which are described and discussed below:

**Figure 1:**
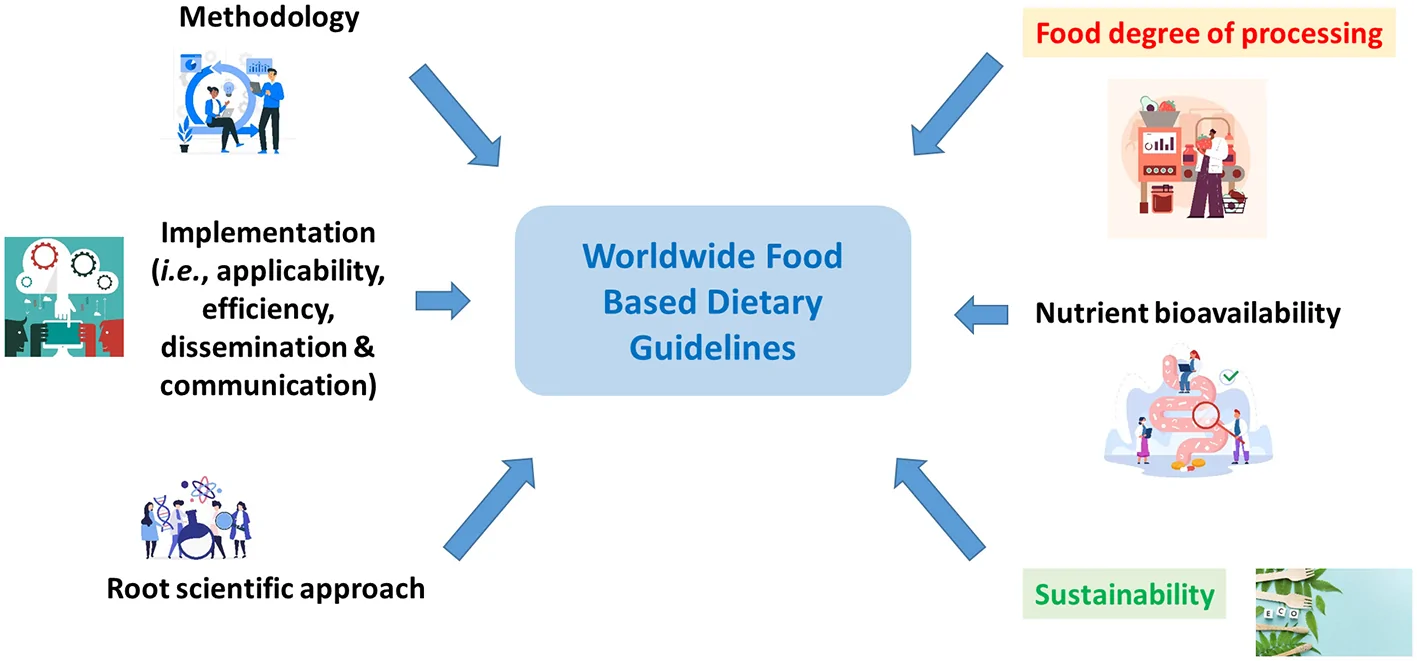
Main general shortcomings of food-based dietary guidelines worldwide.

### 3.1 FBDG methodology

The first emphasized shortcoming concerns the FBDG methodology. According to Zeraatkar et al. (2019), “*A growing body of evidence supports the notion that there are serious deficiencies in the methods used to develop dietary guidelines”*, notably including the difficulty of conducting comprehensive systematic reviews, the heterogeneous evaluation of the quality of the evidence, and the insufficiently strict management of conflicts of interest (12). The authors propose the use of the Grading of Recommendations Assessment, Development and Evaluation (GRADE) approach as the most rigorous and fully developed approach to address these problems, and to substantially improve public policy decision-making (13). However, this approach may also have limitations for nutritional guidelines. Specifically, it tends to undervalue observational studies, which are critical in nutrition research, and may not fully consider contextual factors such as cultural preferences, regional dietary patterns, and socioeconomic conditions.

A notable example of insufficient conflict of interest management can be observed in the composition of the U.S. Dietary Guidelines Advisory Committee (DGAC), where several members have been found to maintain ties with the agri-food industry (14). This conflict of interest may be particularly concerning given that the Committee is responsible for issuing recommendations on topics such as ultra-processed foods (UPFs) and animal-based foods, which constitute a significant source of revenue for major food corporations worldwide (15). In addition, the last USA FBDG is the only one that does not address the issue of the sustainability of animal products (16), while, paradoxically, recommending to significantly reduce the consumption “highly processed foods”. In fact, both UPFs and animal-based foods consumption should be reduced for a more sustainable global health. Such entanglements may undermine the perceived impartiality and scientific integrity of FBDGs and raise broader concerns regarding the credibility and independence of future guideline development processes.

### 3.2 FBDG implementation

Little research has been conducted to analyse whether FBDGs are utilized by the public and to evaluate the efficacy of FBDGs as a public health promotion tool. In fact, despite years of existence, FBDGs do not appear effective at changing consumer behavior to reduce the prevalence of chronic diseases (17). Several explanations for this observation are proposed, including a lack of political support and stakeholder engagement, conflict with marketing campaigns during FBDG development and implementation, uncertainty in both the nutritional and social sciences relative to the complexity of the diet–chronic disease relationship, and/or the multifactorial origin of chronic diseases, which includes critical factors other than food (17).

Several dietary indices have been used to measure compliance with FBDGs, *e.g*., for WHO guidelines (18), in China (19), Spain (20), France (21), and Brazil (22), notably the recent BraScore including the recommendation to avoid UPFs (23). These evaluations generally revealed that compliance has a large margin for improvement. For example, a low degree of compliance with FBDGs was evaluated in 4,371 participants in Switzerland; consequently, the goal was to continue to inform the population about healthy and balanced diets (24). However, compared with men, women had better compliance with FBDGs regarding the consumption of fruits, vegetables and meat, as observed in other European studies. Other parameters, such as a younger age, overweight/obesity status, smoking status and educational status, are associated with lower adherence to FBDGs. As proposed, the focus of policies would need “*to move beyond merely disseminating FBDGs*” and “*be part of a wider public health nutrition strategy involving multiple sectors and policy levels*” (25).

With respect to FBDG communication, key themes related to the effective communication of FBDGs were notably reported in the UK, such as the way in which messages relating to nutrition and sustainability are delivered, the way in which specific population segments are targeted, the ways in which the barriers to and benefits of adopting FBDG are addressed, the development of pragmatic tools and resources to support FBDG implementation, and the better exploitation of social media and marketing techniques to increase public engagement (26). Notably, multi-level strategies were initially proposed in the National Food Strategy for a more sustainable UK food system, but this was not adequately addressed in the 2022 Government Food Strategy.

Following a cross-sectional audit of the FBDGs (*n* = 53 countries listed on the FAO website, mostly high-income and upper-middle-income countries), dissemination strategies and plans included in the international FBDGs, the authors concluded that more research is needed “*on how to improve FBDG dissemination and implementation*”, not only for the larger public but also for “*health and non-health care professionals, as well as other sectors where these guidelines are likely to influence practice*” (27).

In summary, FBDGs have not been sufficiently effective to date in terms of (1) limited and isolated implementation (*i.e*., not involving a sufficient number of stakeholders/interest groups and the lack of development of practical tools and resources), (2) lack of political support, and (3) insufficient communication to drive adherence (as demonstrated in particular in the UK). More generally, literature globally recognizes the importance of coordinated national implementation strategies as these may be more effective than isolated actions (25, 28–30). Implementation research has already been conducted in the context of the Brazilian FBDG - the first to incorporate the Nova classification and broader aspects of healthy and sustainable food systems - describing coordinated implementation actions across different levels of government (28) and playing a crucial role in informing public policies (31). The Brazilian example could help and inspire decision makers in other countries to carry out holistic implementation in an integrated manner.

### 3.3 Scientific approach: reductionist *vs*. holistic paradigms

For more than 20 years, a significant discrepancy has been observed between the increasing global prevalence of chronic diseases and the large body of research on preventive nutrition and human health, which informs FBDGs. Where does the problem come from? Is it consumers who do not follow FBDGs or are the FBDGs not sufficiently well designed by researchers and other specialists and experts? On the one hand, it may be questioned about how we, as researchers, were leading nutritional research and designing FBDGs, and concluded that academics have also a responsibility in addition to consumers and populations (5, 32). One of the main changes would be essentially to move further toward a more prominent empirico-inductive (*i.e*., the research begins in an exploratory manner with the researcher's experience), qualitative and holistic approach in food and nutrition research—food and nutrition sciences being first holistic sciences by essence (32–34). This approach involves developing unifying concepts derived from data collected in real life and anchored with the reality of consumers, rendering them easier to appropriate (34). Thus, through the application of this approach new qualitative and unifying concepts have been developed: (1) the preservation of the food “matrix effect” as a core paradigm for elaborating FBDGs before food groups and the nutrients they should theoretically supply; (2) the 3V and subsequent 4V rule for healthy and sustainable diets (see Section 2.5.3) (35–37); (3) food scoring based on the degree of processing (38) with an emphasis on UPFs as a holistic target for FBDGs for consumers (5, 37); and (4) the identification of excessively degraded and/or artificialized food matrices as main contributors to the increased prevalence of chronic diseases (39).

However, combining both holistic and reductionist approaches is necessary, but always beginning with holistic approaches, *i.e*., from the whole to the specific/parts in a hierarchical manner (36). Moreover, ultra-reductionist, nutrient-centered food scoring systems may unintentionally favor the interests of the agri-food industry rather than supporting broader public health objectives, *e.g*., as shown by the Australian Health Star Rating system, which favors the use of non-nutritive sweeteners to obtain better scores and leads to more UPFs (40), or with French Nutri-score scoring high a substantial percentage of unhealthy UPFs (41). These findings highlight the need for more holistic scoring systems that consider food structure and processing levels, especially since people consume complex foods, not isolated nutrients, harmoniously combined with more nutrient-centered criteria, *e.g*., added alt and/or sugars. In this way, food composition databases are still too reductionist and provide little information about the real health potential of food (42).

Consistent with our studies related to holistic and reductionist approaches in food and nutritional sciences, Phelan et al. advocated the need to include “*contextual factors when seeking to understand the barriers and facilitators for optimizing dietary quality*” and proposed a holistic approach to assessing dietary quality (43). Previously, Parker et al. also advocated for more holistic FBDGs encompassing both environmental sustainability and human health to harness synergistic solutions through common causes—free of conflicts of interest—and to promote local products to support this goal (44). They suggested that FBDGs should “*act as a policy springboard for effective regulatory action to change the food environment and not just as an education tool”*. As a complement to the reductionistic tendency, the holistic approach is also supported by Baltussen, who emphasizes that (1) multiple other aspects, in addition to nutrients and calories, explain the properties of foods and their effects on the body (*e.g*., food processing, food matrix, food combinations, individual effects, food taste and food digestion), (2) nutrients and calories are not interchangeable from one food to another, (3) reductionism tends to simplify reality, which has consequences for the complex concept of health (45), and (4) reductionistic knowledge seems to have a limited ability to promote health and prevent multifactorial chronic diseases (46). Notably, “*holism studies health and disease in daily life context, which increases the generalizability of research*” (46); hence, this approach will be more impactful for FBDGs.

Finally, by conducting a survey of 27 countries, researchers concluded that “*FBDGs are generally not utilized to their full potential*” (30); and the authors propose a more holistic approach that involves other sectors in FBDG development and implementation in addition to the nutrition and health sectors, *i.e*., a food systems approach beyond health and education.

### 3.4 Consideration of nutrient bioavailability

This shortcoming was the least mentioned in the literature, except in a recent paper (47). The main idea is that, depending on the food sources, the same nutrient is not equally bioavailable, which is not considered in FBDGs or in sustainability measurements. The authors concluded the “*importance of developing nutrient bioavailability as a requisite nutrition metric to contextualize the environmental impacts of different diets*”. However, as we already concluded in 2016 for the updating of food consumption benchmarks with French ANSES (*i.e*., Agence Nationale de Sécurité Sanitaire de l'Alimentation, de l'Environnement et du Travail) in view of updating the new French PNNS n°4 (2017–2021), the literature about vitamin and mineral bioavailability in humans for each food or food group is currently largely insufficient to correctly implement this point in FBDGs, notably because of the limited number of high-quality studies, and notably being very expensive to conduct (48). Therefore, this issue is very unlikely to be seriously considered in the future FBDGs.

### 3.5 Actionable recommendations: Considering the degree of food processing and sustainability in FBDGs

Two important limitations of the current FBDGs concern the insufficient consideration of food processing and sustainability aspects. Previous work, including our 2015 position paper co-authored with international researchers involved in the development of the Nova classification, highlighted that most dietary recommendations and epidemiological studies still focus primarily on nutrients and food groups but largely overlook the degree of food processing (5). This oversight can lead to inconsistent or misleading recommendations, as foods within the same food group may differ substantially in their health effects depending on their processing level.

Similarly, several recent analyses have shown that sustainability considerations remain only weakly integrated into FBDGs worldwide (49, 50). Although progress has been made in some countries, environmental dimensions are often insufficiently addressed alongside health objectives.

These two issues—food processing and sustainability—are now discussed in more detail in the following sections.

#### 3.5.1 Global analysis of environmental sustainability in national FBDGs

Numerous scientific studies have emphasized the need to consider sustainability issues more in FBDGs. A short review of these papers and analyses, from 2021 to 2024, with the main conclusions is presented below, first, a global analysis, followed by a focus on specific countries. Studies published before 2021 confirm the analyses and conclusions described below, but they did not introduce any new important ‘guiding principles' for including sustainability in FBDGs.

As stated by Van Dooren et al. (2024), “*For many decades, FBDGs were only health oriented. This changed post-2009 when gradually, an increasing number of countries began to include environmental sustainability considerations in their guidelines*.” (49). International organizations such as the Food and Agriculture Organization (FAO) and the World Health Organization (WHO) asked governments to include environmental sustainability in future FBDGs (51). Van Dooren et al. also observed that incorporating environmental sustainability into FBDGs represents a policy innovation, reflecting an ongoing debate between traditional guidelines and new multicriteria approaches to healthy eating (49). They also noted the growing consensus that changes in the food system are both inevitable and necessary, while highlighting that “*methodologies on how this should be done are lacking*”.

Several reviews have explored how sustainability is included in FBDGs worldwide. The most recent and comprehensive analysis of this topic is the study by James-Martin et al. (50). Among the 95 countries with FBDGs identified by the FAO, 83 countries were assessed based on inclusion criteria (based on the FAO's guiding principles for sustainable healthy diets (51)). Of these guidelines, 37 mentioned environmental sustainability. The results indicated that sustainability was included only in a few FBDGs, and that the topics of “*how dietary changes can be made*” or of “*quantified advice for implementing sustainable diets*” were not considered thoroughly, with great variations observed among FBDGs. Common principles address culture, the inclusion of more plant-based foods, environmental effects, biodiversity, and food waste.

Using the same approach, Martini et al. collected 43 FBDGs from the online repository developed by the FAO and analyzed them for their adherence to each of the guiding principles (52). The authors reached similar conclusions, notably that health outcomes were prioritized and that current FBDGs are “*poorly aligned with the guiding principles*” for sustainable healthy diets proposed by the FAO and the WHO. However, Europe appears to be the global region that has the highest rate of inclusion of guiding principles for sustainable healthy diets related to environmental impact, but with no mention of the impact of food processing on environment (see below Part 2.5.2.2). The authors also emphasized the need to improve “*knowledge among the general population on this topic, as well as in improving dietary behaviors*”.

In the same approach, Fabri et al. analyzed FBDGs from 90 different countries and reported that less than half (*n* = 40) of the FBDGs addressed sustainable or symbolic aspects (*i.e*., linked to traditions/culture) of food, and only 13 FBDGs addressed both aspects (53). Another analysis of 85 countries suggested that FBDGs could be both healthier and more sustainable, notably by limiting the consumption of animal-based foods, especially beef and dairy (54). All national FBDGs, on average, exceed environmental limits, particularly for greenhouse gas (GHG) emissions (well over 200% of the target value) and nitrogen and cropland use (both near or above 100%), while the WHO and EAT-Lancet dietary guidelines are closer to target values (Figure 2).

**Figure 2:**
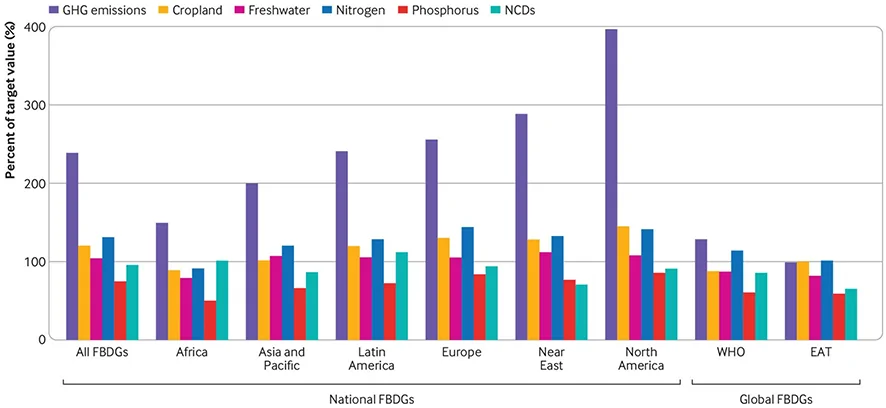
Comparison of the health and environmental impacts of universally adopting food based dietary guidelines (FBDGss) to a set of global health and environmental targets. Targets include the sustainable development goal of reducing premature mortality from non-communicable diseases (NCDs) by a third, the Paris Climate Agreement to limit global warming to below 2 °C (greenhouse gas (GHG) emissions), the Aichi biodiversity target of limiting the rate of land use change (cropland), and the sustainable development goals and planetary boundaries related to freshwater use, and nitrogen and phosphorus pollution. Estimates are expressed as percentage of attained target value averaged across countries in FBDGs regions. Values of 100% or less indicate that environmental and health impacts are in compliance with the targets, and values greater than 100% indicate that targets are exceeded. WHO=World Health Organization; EAT = EAT-Lancet Commission on Healthy Diets from Sustainable Food Systems. Reproduced from Springmann et al. (54), licensed under CC BY 4.0.

In addition to these comprehensive analyses of FBDGs worldwide, Mazac et al. investigated “*how and why concepts related to sustainability were included or excluded* [in FBDGs]”, adding that “*such motivations behind concept inclusion are important for developing policy approaches which include sustainability domains in FBGDs in the future*” (55).

More quantitatively, Cambeses-Franco et al. (2022) modeled and compared the main environmental indicators in terms of the carbon footprint and water footprint and nutritional quality (*via* the Nutrient Rich Diet index, NRD9.3, and a health gain score) of northern/southern European and American FBDGs (56). Notably, they reported that adherence to the American Dietary Guidelines (USA) was associated with higher carbon emissions, *i.e*., “*more than 1.3 times higher when the comparison was made with the most plant-rich European diets analyzed*” (*e.g*., Italian FBDGs and Mediterranean diet). This difference can be attributed to the higher quantities of dairy products and meat in American Dietary Guidelines. However, more generally, greater adherence to the recommended FBDGs was associated with benefits for both nutritional and environmental indicators.

Although some countries have already integrated sustainability criteria into the modeling of their FBDGs (*e.g*., Germany (57) and The Netherlands (58)), the compliance of diets with FBDGs and other sustainable dietary models has been assessed in some cases, sometimes leading to country-specific conclusions, *e.g*., Nordic countries (59), Italy (60), UK (61), Switzerland (61), Australia (62), Iran (63) and China (64), or a more recent global model (65).

In conclusion, a relevant margin appears to exist for increasing the sustainable potential of FBDGs worldwide. However, they should be implemented depending on regional specificities, such as culinary habits and/or local production. Actually, this is what is proposed by the EAT-Lancet Planetary Health Reference Diet 2.0 (66), that has to be adapted to national contexts, rather than adopted uniformly across countries (see also 2.5.3 for the 4V rule).

#### 3.5.2 Discussion of the inclusion of the degree of food processing in FBDGs

##### 3.5.2.1 Food processing and human health

The link between food processing and human health has been reviewed in a previous study (67). Briefly, in addition to ultra-processing, the main studied technological processes that may negatively impact human health are the pressing of fruits into juice, the processing of meats and the refining of cereals. No comprehensive epidemiological studies or subsequent meta-analyses have been conducted on many other processes (67).

Since the introduction of the UPF concept by Monteiro in 2009 (68), scientific interest has grown rapidly. As of June 2026, the body of research includes at least 310 epidemiological studies (both cross-sectional and longitudinal) relating UPF consumption to chronic diseases and/or early mortality risks, including at least 138 longitudinal prospective cohort studies (*i.e*., 45% of all epidemiological studies), more than 100 narrative or systematic reviews and/or meta-analyses regarding the association of UPFs with human health, and at least 13 interventional studies.

An increasing number of these studies suggest not only strong statistical associations but also emerging evidence of causal links between high UPF consumption and adverse health outcomes (69). For example, a recent umbrella review of epidemiological meta-analyses by Lane et al. (70) provided the most convincing associations of UPF consumption with mental health, wheezing, all-cause mortality and type 2 diabetes. Other health outcomes warrant further prospective and longitudinal cohort studies to reduce statistical heterogeneity, notably because of differences in the size and characteristics of studied populations and/or the number of studies considered for a given health outcome, among other factors.

Concerning the risk of all-cause early mortality, the simulated dose–response risk (71) is worth mentioning, which shows a linear and significant increase in risk for each additional 5% of the calories derived from UPFs. While other simulated dose–response analyses related to other chronic diseases are awaited, a recommendation *(i.e*., precautionary principle) “not to exceed ≈ 10%−15% of daily calories from UPFs”, which corresponds to approximately 1–2 servings/day, could be promoted.

In conclusion, processed meat (72) and UPFs (which often include processed meat, though not all types) (69) appear to be the most detrimental forms of food processing to human health - more so than fruit juice and refined cereals - due to their association with a significantly increased risk of several chronic diseases. Therefore, the distinction between processed foods and UPFs should be made clear, as first done by the Nova classification (73).

More recently, in addition to the relationship between food processing and health, another research topic has begun to emerge through the study of food (ultra)processing with respect to food system sustainability and the environmental impacts (74–76).

##### 3.5.2.2 Food (ultra)processing and planetary health

Although UPFs were first linked to sustainability in the 2014 Brazilian FBDGs (77), the environmental implications of UPFs are still under investigation, especially to generate more quantitative data. The initial main studies addressing this issue have been previously reviewed and synthesized (37). Beyond this short review, a new and more recent large-scale study based on the European EPIC cohort (n = 368,733 participants) reported that “*UPF consumption was strongly associated with GHGe and land use as compared with unprocessed or minimally processed food consumption*”, and the authors further proposed substituting UPFs with unprocessed or minimally processed foods to achieve a reduction in GHGE and land use of approximately 9% (78). In addition, UPFs prompt individuals to eat more than needed (79, 80), leading to the potential production and intake of unnecessary surplus calories (81), which may ultimately strain both human health and planetary boundaries, and prevent them from needing to be replaced by other foods; *e.g*., obesity is associated with 1.6% of worldwide GHGEs (82).

Therefore, the UPF issue is global and holistic and extends beyond its risks to human health (37). Thus, while UPFs address food availability, convenience, and safety well, these benefits often come at the expense of environmental sustainability and socioeconomic resilience (74). Thus, an analysis by an international consortium suggests that food processing should be more aligned with circular economy principles and regional food systems (74). These principles involve favoring minimal processing techniques that preserve the natural food matrix rather than relying on reductionist, siloed innovations that often ignore the broader sustainability context, *e.g*., insect proteins, *in vitro* meat, or ultra-processed plant-based meat alternatives (74). Other researchers have begun to conceptualize the drivers of UPF production and consumption, along with their environmental impacts, as a basis for future studies to support this shift (75).

Beyond ultra-processing, other processing technologies have been studied for their impacts on food system sustainability. Sovacool et al. proposed 78 commercially available, emerging, and experimental innovations for decarbonizing the food and beverage industry and reported that dehydration processes are particularly demanding in terms of energy use (83). Otherwise, with a holistic approach, the Barilla Foundation in collaboration with the UN Sustainable Development Solutions Network (UN SDSN) proposed four pillars for agro-food industry sustainability to align with the Sustainable Development Goals (SDGs), *i.e*., (1) beneficial products and strategies contributing to healthy and sustainable diets, (2) sustainable business operations, (3) sustainable supply and value chains and (4) good corporate citizenship (84). However, among the 100 worldwide agri-food companies analyzed in this report, none address the four pillars, and real firms' contributions to sustainable food systems are still unclear.

Overall, food industries are neglected but major actors of the agri-food chain, driving upstream the quality of food production and downstream the health quality of final products; therefore, a more sustainable agri-food industry may drive upstream more sustainable food production that potentially might emit less greenhouse gases than it currently does (74).

##### 3.5.2.3 The inclusion of the degree of food processing within existing FBDGs

###### 3.5.2.3.1 Up to the advent of the Brazilian guidelines

Before the advent of the UPF concept, food processing was only slightly and indirectly considered in FBDGs (5), *e.g*., wholegrain *vs*. refined cereals, whole *vs*. semi-skim *vs*. skim milk, red *vs*. processed meat, and raw fruit *vs*. fruit juices and sweetened fruit juices/sodas (67, 85). In addition, the usual recommendation to limit the consumption of discretionary/non-core foods, *i.e*., products that are too sweet, salty and/or fatty, is generally given at the top of most food pyramids, but the advice remains vague for consumers and does not include all unhealthy industrial foods, *e.g*., diet/light soda. In addition, a dried fruit contains fivefold more sugars per 100 g than a regular soda, although it is not unhealthy, while sweetened beverages are associated with increased risks of some chronic diseases (86), but not dried fruits (85, 87). This finding reflects the differential health effects of natural *vs*. free sugars (39).

In fact, at each level of the food pyramids, although raw and minimally processed foods are generally graphically represented, an uninformed consumer could choose UPFs that are not necessarily healthy, *e.g*., at the first level of fruits and vegetables, an industrial fruit juice rich in free sugars or an industrial ready-to-eat vegetable soup; at the second level, a cooked–extruded sweetened breakfast cereal; at the third level, a flavored yogurt with added sugar; at the fourth level, ultra-processed deli meat with nitrites; and, finally, at the last level, ultra-processed spreads and refined oils. This finding may be explained by the first American food pyramid that was developed in 1992, which was built to reflect first nutrient supplies, not the degree of processing, and was derived from a nutrient-centered and reductionist scientific approach: fruits and vegetables for fiber, minerals and vitamins; cereals for fiber and complex carbohydrates; fishes, meats, eggs and dairy for proteins; and spreads, butter and oils for lipids (88). One hypothesis as to why highly processed foods are not yet included in FBDGs could be the lack of a systematic consideration of the degree of food processing in epidemiological studies, which in turn could explain some of the contradictory results that are often reported between certain food groups and the risk of chronic diseases (89).

Therefore, a more systematic consideration of food processing in FBDGs is quite recent and started with the Brazilian Food Recommendations published in 2014 (77). These recommendations followed the publication of the Nova classification in 2010 in its first format (three technological groups), followed by the final four technological groups in 2013–2014, *i.e*., un/minimally processed foods, traditional processed culinary ingredients, processed foods, and UPFs (90). The 2014 Brazilian FBDGs are noteworthy for three main reasons: (1) they are not reductionist and nutrient-centered, and focus primarily on foods rather than nutrients; (2) they adopt a holistic and qualitative approach, recommending the consumption of fresh, unprocessed or minimally processed foods, cooked meals and shared meals, *i.e*., not UPFs; and (3) they integrate the degree of food processing into their framework, notably using a graphical representation with three levels, *i.e*., natural food, processed food and UPFs, for each food group.

Using this approach, these guidelines attempted to respond to several of the abovementioned shortcomings identified in earlier FBDGs, particularly in terms of their holistic and food-based orientation. Therefore, they have been discussed as a potential model for the development of future FBDGs in other countries, provided that they are adapted to local culinary traditions and eating habits. In many Western countries, a significant portion of the population may lack the time, resources, or cultural framework to rely predominantly on raw or minimally processed foods in daily life. Therefore, the inclusion of high-quality, nutritionally adequate industrially processed foods may also remain necessary to ensure that dietary recommendations are both practicable and accessible. However, beyond this observation, it is worth mentioning that the erosion of traditional dietary patterns and culinary practices may itself represent a major barrier to returning to diets based predominantly on unprocessed or minimally processed foods. Likewise, constraints related to time and resources could also be discussed from a public policy perspective, highlighting the role of coordinated government actions and broader societal strategies to support healthier food environments, rather than placing the responsibility primarily on individuals. Notably, as previously reported, cooking should be strengthened beyond the strictly domestic sphere, which could “*represent an important strategy for promoting healthier diets while advancing gender equity in contemporary society*” (91).

###### 3.5.2.3.2 The use of the (ultra)processed food concept in other food-based dietary guidelines

Recent reviews have increasingly examined how the concept of food processing—and particularly ultra-processed foods (UPFs)—has been incorporated into Food-Based Dietary Guidelines (FBDGs). Four main themes emerge from this literature: (1) the growing inclusion of food processing in national FBDGs, (2) the predominance of nutrient-centered recommendations despite this development, (3) challenges related to terminology and consumer understanding, and (4) stakeholder perspectives on incorporating food processing into public policy.

####### 3.5.2.3.2.1 Increasing inclusion of food processing in FBDGs

Several reviews demonstrate that food processing has only recently become an explicit component of FBDGs. An analysis of 96 national FBDGs identified recommendations related to the food processing in only 21 guidelines (21.9%), most of which were published after 2012 (Table 1) (92). Similarly, Koios et al. analyzed 106 FBDGs (93), while Anastasiou et al. reviewed 148 guidelines from 96 countries (94). Together, these studies show that although the inclusion of food processing concepts is increasing, it remains limited globally.

**Table 1:** Characterization of the food guides analyzed and food processing level recommendations (*n* = 96). Reproduced from Cintia Cassia Tonieto Gris et al. (92), licensed under CC BY 4.0.

Guides' characterization	Total		Include processing-level recommendations	
	*n*	%	*n*	%
Year of publication				
Before 2005	10	10.42	0	0.00
2006–2011	27	28.13	5	23.81
2012–2017	46	47.92	10	47.62
2018–2021	13	13.54	6	28.57
Total	96	100.00	21	100.00
Place of publication				
Europe	34	35.42	8	38.10
Latin America and the Caribbean	29	30.21	8	38.10
Asia and Pacific	18	18.75	2	9.52
Africa	7	7.29	2	9.52
North America	2	2.08	1	4.76
Middle East	6	6.25	0	0.00
Total	96	100.00	21	100.00

Europe and Latin America currently include the largest number of FBDGs containing recommendations related to food processing (Table 2). Notably, Brazil, France, Belgium and Ecuador explicitly use the term *ultra-processed foods (UPFs)*, indicating growing institutional recognition of the NOVA classification and its relevance for dietary guidance (73). However, whether the inclusion of UPFs in national FBDGs ultimately translates into measurable changes in dietary behavior or health outcomes remains to be evaluated.

**Table 2:** Characterization of information on foods with different processing levels in the main messages of the 21 Food Guides, 2022. Reproduced from Cintia Cassia Tonieto Gris et al. (92), licensed under CC BY 4.0.

Country	Year of the last issue	Terms adopted	Consumption guidance	Coverage	Justification	Exemplification	Guidance on consumption amount
Romania	2006	Highly processed foods	Eat with moderation	Highly processed sugar-rich foods	–	–	–
Austria	2010	Processed foods	Should be consumed with moderation	Processed fat-sugar-and-salt-rich foods	X	X	X
Saint Kitts and Nevis	2010	Processed foods	Use less processed foods	Processed foods in general	–	–	–
Slovenia	2011	Processed and preserved foods^*^	Limit intake	Processed and preserved foods, in general	–	–	–
India	2011	Processed foods	Reduce use	Processed fat-sugar-and-salt-rich foods	–	–	–
El Salvador	2012	Highly processed products	Avoid	Highly processed products, in general	–	–	–
Cyprus	2013	Highly processed products	Limit intake	Highly processed foods in general	X	–	–
Brazil	2014	Minimally processed food; processed foods; ultra-processed foods	Make natural or minimally processed foods the basis of your diet; Limit the consumption of processed	Foods in general	–	–	–
			foods; avoid the consumption of ultra-processed foods	–	–	–	–
Colombia	2014	Processed meat	Reduce the consumption	Processed meat	X	–	–
Former Yugoslav Republic of Macedonia	2014	Highly processed foods	Limit the consumption	Highly processed foods in general	–	–	–
Afghanistan	2015	Highly processed foods	Eat less	Highly processed foods in general	–	–	–
Benin	2015	Highly processed products	–	Processed foods in general	X	–	–
Jamaica	2015	Processed foods	Reduce the intake	Processed foods in general	–	–	–
Kenya	2017	Non-processed food	Include	Non-processed starchy foods	–	–	–
Belgium	2017	Ultra-processed products	Choose as little as possible	Ultra-processed foods in general	–	–	–
Poland	2019	Processed foods; Processed meat	Consume less and replace	Processed foods, in general, and processed meat	–	X	–
Ecuador	2020	Ultra-processed foods	Avoid the consumption	–	X	–	–
Panama	2019	Processed products	Limit the consumption	Processed sodium-rich products	–	X	–
Peru	2019	Processed foods; ultra-processed foods	Reduce the consumption of processed foods; avoid ultra-processed foods	Processed and ultra-processed foods in general	X	–	–
Canada	2019	Highly processed foods	Limit	Highly processed foods in general	–	–	X

^*^Due to the numerous possibilities of interpretation, the term “preserved” was not counted as a “new term” related to the level of food processing.

####### 3.5.2.3.2.2 From nutrient-centered to processing-based recommendations

Despite this recent progress, nutrient-centered recommendations continue to dominate FBDGs worldwide. Across the reviewed guidelines, almost all continue to encourage or discourage foods primarily through their nutrient composition—for example, by promoting vitamins or discouraging salt, sugar and saturated fat—rather than considering the degree of food processing (93).

Although recommendations to limit added sugar, salt and fat remain scientifically justified (95, 96), several authors argue that relying exclusively on nutrient-based advice may create inconsistencies. Many foods discouraged in FBDGs are indeed ultra-processed, yet some minimally processed foods are also discouraged, highlighting potential contradictions between nutrient-based and processing-based approaches. Recent evidence further suggests that food processing itself may contribute independently to adverse health outcomes, supporting the inclusion of processing alongside traditional nutrient-based recommendations (93, 97).

####### 3.5.2.3.2.3 Terminology and consumer understanding

Another recurring finding concerns the considerable variability in terminology used to describe food processing. Terms such as “highly processed”, “ultra-processed”, “processed”, “minimally processed”, and “nonprocessed” are often used inconsistently across national FBDGs (Table 2) (92, 94). This lack of harmonization may complicate comparisons between guidelines and create confusion among consumers.

Several studies therefore emphasize that the successful integration of food processing into FBDGs depends not only on scientific evidence but also on effective communication. Koios et al. concluded that consumer understanding of processing-related terminology and their capacity to act upon such recommendations are essential for successful implementation (93). Quinn et al. similarly found that the diversity of terms used in national FBDGs results in multiple interpretations of the concept of ultra-processing (97). Kraak further highlighted the importance of improving how food processing concepts are communicated to nutrition professionals and consumers, arguing that dietary advice should encourage the selection of minimally processed foods (98).

These findings suggest that future FBDGs should not only incorporate food processing more systematically but also provide clear, consistent terminology accompanied by appropriate education and communication strategies. The Brazilian FBDGs provide one example of how this transition may be achieved (77).

####### 3.5.2.3.2.4 Stakeholder perspectives

Finally, the incorporation of food processing into FBDGs has also generated debate among stakeholders. An evaluation of the development of the Brazilian Food Guide identified two conflicting perspectives (99). One view that “*aligned with the interests of the food industry”*, which was supported mainly by the food industry itself and food organizations/unions, and another view “*of healthy eating, which serves the interests of the population*”, which was supported mainly by organizations related to food and nutrition security, non-profit organizations, and the government (99). In the first view, stakeholders were against the adoption of a food processing-based classification system in a public policy such as the new Brazilian Food Guide (77). In contrast, the second group supported the general orientation of the new food guide but criticized it for its description of some important issues, *e.g*., pesticides, GMOs and organic food; food advertising; and the lack of discussion related to existing nutrition and food policies in Brazil.

#### 3.5.3 The 4V rule: an attempt to address the shortcomings of FBDGs while integrating the degree of food processing and sustainability

Several modeling exercises have therefore been proposed to integrate the degree of food processing and sustainability in FBDGs, but many more have integrated sustainability. In 2014, the Brazilian Dietary Guidelines were the first to integrate the UPF concept (77), followed by several other countries (see Table 2 for the list), *e.g*., France in 2019 (100). With respect to the inclusion of sustainability and environmental impacts, other innovative approaches have been explored to improve FBDGs through the integration of sustainability criteria (*e.g*., Dutch and German modeling exercises). The FBDG in Germany was based on the definition of sustainable nutrition according to the Scientific Advisory Board of the Federal Ministry (WBAE), which integrates four key objectives: health, social aspects, animal welfare, and the environment (101). In the Netherlands, scientific expert groups with expertise in the fields of nutrition, behavior and sustainability were involved in the development of the FBDG. Notably, the environmental impact was considered by setting maximum levels for foods from animal origin with high greenhouse gas (GHG) emissions (58).

Besides, in our previous works, we proposed a holistic, simple and hierarchized framework for potential future and forwards-looking FBDGs that is easy for consumers to understand. This framework was proposed to address the shortcomings of FBDGs while integrating the degree of food processing and sustainability in a hierarchical manner. Thus, we developed the 3V rule (35), followed by the 4V rule, *i.e*., Veritable, Vegetal, and Varied foods, and “Vivant” (“Living” in English) for regenerative agriculture that protects the living (102) (Figure 3). The Veritable metrics address the degree of food processing, the Vegetal metrics address the plant/animal ratio, and the Varied metrics address food diversity. This generic and simple rule is a qualitative, holistic and unifying concept based on an empirico-inductive approach (34). It was developed in four steps: (1) the identification of the three interconnected dimensions characterizing the diet–human health relationship (36); (2) the elaboration of a threshold for each dimension in terms of calories consumed daily for UPFs and animal-based foods, and the number of foods consumed/week for diversity (detailed sources and calculations can be found in the study by Fardet and Rock, 2020 (35); (3) the identification of a hierarchical application of the three first dimensions (39); (4) and the addition of the fourth ‘V' for the quality of production (Vivant, *i.e*., living), and to more generally characterize the diet–global health relationship (102), notably through agro-ecological agricultural practices which can be theoretically extended on a European scale at horizon 2050 (103). However, adapting the 4V decision tree to each country or region by considering regional or local culinary traditions, pedoclimatic conditions, etc., is advised. Finally, the 4V rule also represents a simple tool for the education of people of various ages and populations. In practice, when food is bought, the Veritable rule implies choosing a food based on the quality of the ingredients, *i.e*., avoiding markers of ultra-processing (104), which is not an easy task, and not on food composition, because no food is nutritionally balanced; hence, the recommendation to “*eat varied foods*” (39).

**Figure 3:**
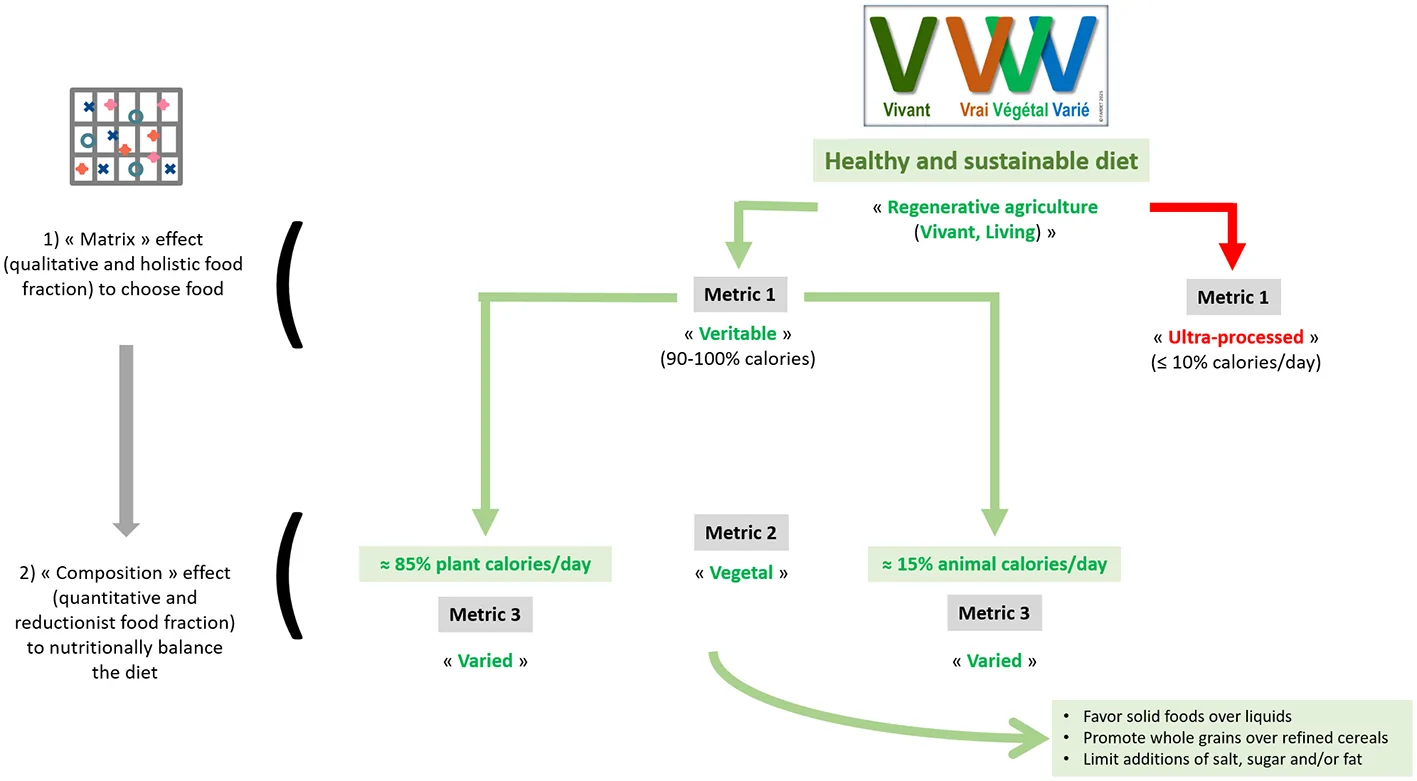
The holistic and unifying 4V rule decision tree to hierarchically integrate sustainability and food processing issues for a healthy and sustainable dietary pattern. Adapted from Fardet and Rock (39), with permission.

## 4 Conclusions and perspectives

Although the inclusion of the sustainability issue in FBDGs has now reached a consensus, the integration of the degree of food processing has not. Our scientific analysis is that strong scientific evidence that food processing plays critical roles in human and planetary health is now available. This dimension may represent the missing link for achieving a more holistic understanding of the relationship between diet and health outcomes. While further research is obviously necessary, the current scientific evidence appears sufficiently robust to warrant the inclusion of food processing levels in future FBDGs as a precautionary measure, focusing not only on UPFs but also on other technological treatments, *e.g*., refining cereals, pressing fruits, or fermenting vegetables. Failing to include food processing risks perpetuating reductionist, nutrient-centered approaches that align—intentionally or unintentionally—with the interests of large multinational agri-food corporations to the detriment of public health and food system sustainability (8). If we currently do not know whether including UPFs in FBDGs may have long-term health effects, we observe that the previous FBDGs—with sometimes a minor focus on the degree of food processing—have apparently failed to prevent chronic disease development worldwide.

With respect to future FBDGs, in addition to reducing the consumption of red and processed meat and salty/sweet/fatty foods, several changing paradigms may be proposed based on previously identified shortcomings to make FBDGs more impactful for consumers' health and environmental preservation, *e.g*., moving from food pyramids to technological pyramids, including more qualitative and holistic recommendations (which are easier to appropriate for consumers), moving from only food groups and food composition to the food matrix as a core paradigm of FBDGs, and integrating more clearly regenerative agricultural practices and the environmental cost of foods (Table 3).

**Table 3:** Propositions of changing paradigms to integrate in food-based dietary guidelines.

Today	Future
Food group-based pyramids indirectly reflecting the supply of nutrients	Three-stage technological pyramids for each food group: (1) unprocessed/minimally processed, (2) processed, and (3) ultra-processed foods or the development of other graphical representations (*e.g*., icons or food guide images) consistent with updated recommendations
Linear information without a clear hierarchy	Hierarchizing food and nutritional information, from the more holistic approach to the most specific terms
Both quantitative (*e.g*., portion sizes and nutrient threshold not to exceed) and qualitative information	Developing more the qualitative aspects, *e.g*., the pleasure of eating, the importance of sharing meals, and cooking at home
A strong focus on food groups and nutrients	Focusing more on the importance of the effect of the food matrix on health, and hence the degree of food processing
Food composition scores are included, which may contradict more general recommendations, *e.g*., the Nutri-score in France and Health Star Rating system in Australia which score as “potentially healthy” many UPFs	Moving to food scores based on the degree of processing
An as yet insufficient focus on the sustainability issue	A stronger focus on the sustainability issue, *e.g*., regenerative agro-ecology practices

Overall, the main challenge for future FBDGs might be to adopt a more holistic and qualitative approach that recognizes the interconnectedness of humans, animals and the environment (61), and to prioritize nutritional information from the most general to the most specific, *e.g*., nutrients are less relevant to sustainability than the overall quality of dietary patterns. Food groups, their degree of processing, and food system sustainability are therefore mutually inclusive, and a holistic approach enables the development of simple, noncontradictory recommendations for healthy and sustainable dietary patterns in each country. The engagement of multiple stakeholders, including individuals involved in food education, politicians, agri-food chain actors, the media, dieticians and health professionals, in supporting FBDGs also appears mandatory to achieve this goal. The engagement of these groups will require us to break and change the paradigms that underpin our approach to nutrition sciences and hence develop new FBDGs.

Therefore, scientific evidence and climate and environmental emergencies should prompt public authorities and FBDG expert committees (without conflicts of interest) to include more information on sustainability and food processing in the framework of future FBDGs.
